# Targeting ZBP1-Mediated PANoptosis: Inflammation-Responsive Selenized Chitosan Nanoparticles Loaded with Moringa A for Antiviral Pneumonia Therapy

**DOI:** 10.34133/bmr.0234

**Published:** 2025-08-05

**Authors:** Wenhui Wu, Ruidong Li, Chunmei Lv, Dandan Yang, Shunqiang Song, Min Yang, Yongai Xiong

**Affiliations:** ^1^ Institute of Chinese Pharmaceutical Preparations, Chongqing Traditional Chinese Medicine Hospital, Chongqing 400021, China.; ^2^ Key Laboratory of Basic Pharmacology of Guizhou Province and School of Pharmacy, Zunyi Medical University, Zunyi, Guizhou 563000, China.; ^3^ Guizhou Provincial Key Laboratory of Innovation and Manufacturing for Pharmaceuticals and School of Pharmacy, Zunyi Medical University, Zunyi, Guizhou 563000, China.

## Abstract

Viral pneumonia poses a major global public health challenge, where excessive inflammatory responses contribute to tissue damage and respiratory failure. Inflammation-responsive nanoparticles can target inflamed areas, improving drug delivery while minimizing side effects. Chitosan, a biocompatible polysaccharide with anti-inflammatory and immunomodulatory properties, gains enhanced antioxidant and anti-inflammatory capabilities when combined with selenium. This study developed selenium–chitosan nanoparticles loaded with Moringa A (MA), a natural antiviral compound from *Moringa oleifera* seeds. These nanoparticles target lung inflammation, releasing MA to suppress viral replication and infection while reducing inflammatory responses. Additionally, selenium–chitosan nanoparticles mitigate oxidative stress, regulate immunity, and inhibit PANoptosis—a cell death pathway that exacerbates inflammation. By blocking core proteins in this pathway, they further curb inflammatory factor release. This approach offers a promising therapeutic strategy for viral pneumonia, combining targeted drug delivery, antiviral action, and inflammation control with reduced side effects.

## Introduction

Viral pneumonia poses a serious threat to public health. It is caused by a variety of viruses, such as influenza virus, respiratory syncytial virus, coronavirus, and so on. It is extremely harmful to infants, the elderly, and people with immunodeficiency [1]. It not only can cause symptoms like fever, cough, and breathing difficulties but also may lead to severe complications such as respiratory failure, liver failure, and heart failure, and even endanger lives. However, current treatments face numerous challenges. On the one hand, new mutant viruses, like severe acute respiratory syndrome coronavirus 2 (SARS-CoV-2), keep emerging, bringing difficulties to prevention, control, and treatment. On the other hand, there is a lack of broad-spectrum antiviral drugs, making it difficult to deal with multiple viral infections. Moreover, existing drugs may have issues such as drug resistance and side effects, and the development of precise treatment plans is hindered by individual differences and viral diversity [2,3]. Research shows that viral infections may trigger an overactivation of the body’s immune system, leading to a pathological state known as a “cytokine storm”. This abnormal immune response causes a large-scale release of inflammatory mediators such as interleukin-6 (IL-6) and tumor necrosis factor-α (TNF-α) in a short period, thereby triggering systemic inflammation. This uncontrolled inflammatory cascade not only directly damages alveolar tissue, leading to the occurrence of acute respiratory distress syndrome (ARDS) but also may affect organs such as the heart, liver, and kidneys, ultimately resulting in multiple organ dysfunction [4,5]. Therefore, while combating viruses, the control of inflammation cannot be ignored.

**Fig. 1.**
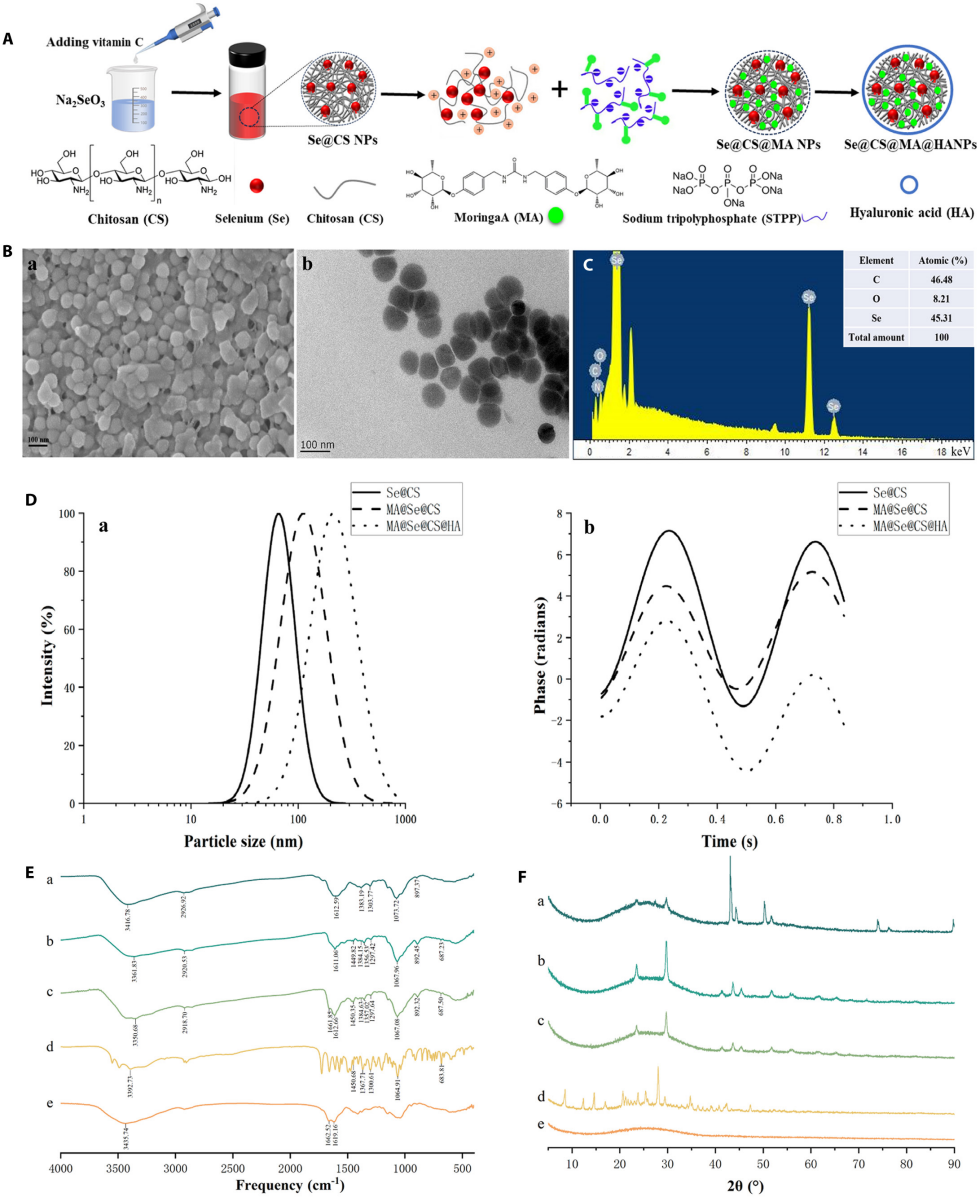
Preparation and characterization of Se@CS@MA@HANPs (Se + MANPs). (A) Schematic illustration of Se + MANPs synthesis. (B) SEM (a) and TEM (b) images of Se + MANPs. (C) Elemental analysis of Se + MANPs. (D) Particle size distribution (a) and potential distribution (b) of Se + MANPs. (E) FTIR spectrum. (F) XRD spectrum.

Moringa seeds are the seeds of the M*oringa oleifera* tree, belonging to the genus *Moringa Adans* of the family Moringaceae. They have extensive edible and medicinal values [6]. In our previous research, we conducted isolation and screening of the active ingredients in Moringa seeds with the guidance of antiviral activity. A novel structured compound with remarkable anti-influenza virus activity was obtained from the acetone-extracted phase of the methanol extract of moringa seeds, and it was named Moringa A (MA) [7]. Its chemical structure is shown in Fig. 1A. Previous studies have found that MA can exert a broad-spectrum anti-influenza virus effect by regulating the autophagy–lysosome pathway, making it a highly promising small-molecule compound against influenza virus [8]. However, the low solubility of MA limits its bioavailability and efficacy. Due to its poor solubility, its absorption in the gastrointestinal tract is restricted, resulting in a reduced amount of the drug entering the systemic circulation and a decrease in bioavailability. At the same time, the low solubility also delays the release and onset time of MA. It may be difficult for the blood drug concentration to reach an effective level, thus weakening the therapeutic effect. To improve its antiviral effect, this project aims to use a chitosan-based nanodelivery system to improve the solubility of MA, enhance its bioavailability, and increase its efficacy. The chitosan nanoparticle drug delivery system can enhance the stability of drugs, control drug release, and improve the cellular uptake ability of drugs. It is an excellent nanodelivery carrier.

Selenium nanoparticles (SeNPs) are a kind of particles with a unique nanostructure. SeNPs can not only effectively scavenge oxygen free radicals but also markedly reduce the secretion level of pro-inflammatory factors such as IL-6 by regulating the activity of immune cells, thus showing a powerful anti-inflammatory effect [9,10]. Its mechanism of action involves activating antioxidant enzymes [such as glutathione peroxidase (GPx)] to enhance the antioxidant capacity of cells and further alleviate the inflammation caused by oxidative stress. Its remarkable efficacy in multiple inflammatory disease models indicates that SeNPs has broad application potential in anti-inflammatory treatment. However, due to its high surface energy and chemical activity, SeNPs are prone to aggregation and oxidation reactions. These reactions can lead to changes in their structure and surface properties, thereby affecting their stability and function [11,12]. This instability may cause SeNPs to lose their unique nanoscale properties during storage or application, restricting their applications in fields such as biomedicine and catalysis. To improve the stability of SeNPs, it is often necessary to protect them through methods such as surface modification, coating, or treatment with dispersants.

To address the stability challenges associated with (SeNPs), we employed chitosan as a carrier matrix for the synthesis of selenized chitosan. This functional composite material integrates elemental selenium with chitosan, effectively combining the inherent biocompatibility and biodegradability of chitosan with the potent antioxidant and anti-inflammatory properties of selenium. Structural characterization reveals that the selenium within this composite exists primarily in covalent or coordination bonding configurations, substantially enhancing both its stability and biological activity [13]. The selenized chitosan not only preserves the antibacterial and tissue repair-promoting characteristics of chitosan but also exhibits significantly improved antioxidant capacity and immunomodulatory functions. Notably, spectroscopic studies demonstrate that selenium is stably incorporated within the polymeric network through coordination complexes. This molecular-level synergistic interaction confers multiple functional advantages, making the composite particularly promising for applications in controlled drug delivery systems and tissue engineering [13].

Hyaluronic acid (HA) is an acidic mucopolysaccharide naturally present in human tissues. It has multiple important physiological functions in the body and is widely used in many different medical fields. From a chemical structure perspective, HA is composed of alternating glucuronic acid and N-acetylglucosamine units. This special molecular arrangement gives it excellent biocompatibility and biodegradability, which provides great advantages when it is used in medical applications such as drug carriers [14,15]. In the field of respiratory diseases, HA has shown potential value in targeted treatment of pneumonia. The core therapeutic mechanism of HA lies in its ability to specifically recognize the abnormally elevated CD44 receptor in an inflammatory state. When pneumonia occurs in the lungs, the expression level of the CD44 receptor on the cell surface increases significantly, and HA can specifically bind to it, providing an ideal target-binding basis for the targeted treatment of pneumonia [16,17].

In this study, selenized chitosan is used as the carrier to encapsulate MA, and then HA is used to modify the surface of the selenized chitosan loaded with MA. A nanotargeted drug responsive to the pulmonary inflammatory microenvironment is designed to deliver MA to the pulmonary inflammatory region, enhance its antiviral pneumonia activity, and integrate the inhibitory effect of nanoselenium on pulmonary inflammatory factors and the antiviral effect of MA. We hypothesized that, on the premise of the specific uptake of selenized chitosan nanoparticles by HA-mediated pulmonary inflammatory cells, the chitosan nanoparticles would be disintegrated within the inflammatory cells, and the MA would be delivered and released into the area with a high concentration of influenza virus to exert an antiviral effect. At the same time, the SeNPs would also be released in the cytoplasm to inhibit the cellular oxidative stress induced by viral amplification, thereby effectively controlling the inflammatory response.

## Materials and Methods

### Materials

MA was synthesized in-house through lab preparation. Sodium selenite was obtained from Merck Life Science Technology Co. Ltd. (catalog no. SLCN8615). Ascorbic acid (catalog no. RH561123), HA (catalog no. RH44689, purity **≥** 95%, molecular weight: 10 kDa), and chitosan (catalog no. RH482374, molecular weight = 200 kDa) with a deacetylation degree of 85.0% (5 to 20 mPa·s, 0.5% in 0.5% acetic acid at 20 °C) were purchased from Guangzhou Ronn Biological Technology Co. Ltd. Fetal bovine serum (FBS) (lot no. 10099-141) and DMEM (Dulbecco’s modified Eagle’s medium) High Sugar Medium (lot no. C11995) were purchased from Gibco. Cellular Protein Extraction Kit (Item No. E-BC-E002) was purchased from Wuhan Eliot Bio-Tech Co. Mice IL-1β (catalog no. E-EL-M0037), TNF-α (catalog no. E-EL-M0048), IL-18 (catalog no. E-EL-M0730), IL-6 (catalog no. E-EL-M0043), IL-10 (catalog no. E-EL-M1210 46), superoxide dismutase (SOD, catalog no. E-EL-H6188), and glutathione peroxidase (GPx, catalog no. E-EL-R2491) were purchased from Elabscience Biotechnology Co. Ltd. 2′,7′-Dichlorofluorescin diacetate (catalog no. ab273640), anti-ZBP1 antibody (catalog no. sc-271483), RIPK3 (receptor-interacting protein kinase 3) antibody (catalog no. sc-374639), anti-MLKL (mixed lineage kinase domain-like) antibody (catalog no. sc-293201), GSDMD antibody (catalog no. se-393581), anti-caspase-7 antibody (catalog no. se-56066), and anti-caspase-3 antibody (catalog no. se-81655) were purchased from Santa Cruz Biotechnology Inc. Anti-NF-κB (nuclear factor κB) p65 antibody (catalog no. ab32536) and anti-GAPDH (glyceraldehyde-3-phosphate dehydrogenase) (catalog no. ab8245) were purchased from Abcam. Horseradish enzyme-labeled goat anti-rabbit immunoglobulin G (IgG) (catalog no. SA0012) and bicinchoninic acid (BCA) protein concentration determination kit (catalog no. PC0020) were purchased from Beijing Solebao Technology Co. Ltd. Lipopolysaccharide (LPS) was purchased from Sigma.

### Virus and cell lines

Influenza A virus A/Puerto Rico/8/34 was obtained from the American Type Culture Collection (ATCC). The mouse lung epithelial cell lines MLE-12 and RAW264.7 were purchased from Shanghai Binsui Biotechnology Co. Ltd.

### Preparation and characterization of HA-modified selenized chitosan nanoparticles loading MA (Se@CS@MA@HANPs)

The preparation of HA-modified selenized chitosan nanoparticles loading MA referred to the preparation method of nanoselenium by previous reports [18,19]. Briefly, under magnetic stirring, 2 ml of a 0.5% (mass fraction) chitosan solution was thoroughly mixed with 2 ml of a 0.1 M sodium selenite (Na₂SeO₃) solution, and the volume was adjusted to 10 ml with purified water. An appropriate amount of a 0.5 M ascorbic acid solution (Vc) was added dropwise to the mixture, and the volume was adjusted to 20 ml with water. The mixture was stirred at a speed of 500 rpm for 60 min and then dialyzed for 48 h using a dialysis bag with a molecular weight cutoff of 8,000 to 14,000 (10,000 kDa). The dialysate was replaced every 12 h. Se@CSNPs were collected and stored at 4 °C for later use. MA (10 mg) was weighed and dissolved in 10 ml of a 0.5 mg ml^−1^ sodium tripolyphosphate (STPP) solution. Then, 3 ml of the abovementioned MA solution was added to the selenized chitosan carrier solution. After stirring at 800 rpm for 30 min, a certain amount of HA solution was added so that the drug accounted for a volume ratio of 1:8 of the total volume. Se@CS@MA@HANPs (abbreviated as MA + SeNPs) were collected and stored at 4 °C for later use. The schematic illustration of MA + SeNPs synthesis was shown in Fig. 1A.

The prepared nanoparticles were characterized by using a laser particle size analyzer, scanning electron microscope (SEM), transmission electron microscope (TEM), Fourier transform infrared spectroscopy (FTIR), and x-ray diffraction (XRD). Besides, the drug loading (DL) and encapsulation efficiency (EE) of MA + SeNPs were determined. Briefly, the MA + SeNPs solution was centrifuged at 9,000 rpm for 10 min. The supernatant was collected and filtered using a 0.22-μm microporous membrane. DL and EE were measured using high-performance liquid chromatography (LC2030C3Dplus, SHIMADZU, Japan), according to the chromatographic condition as we set before.

### Determination of cumulative release rate

In this experiment, the dialysis method was used to conduct in vitro drug release studies. The temperature of the water-bath shaker was set at 37 °C, and the oscillation frequency was maintained at a constant speed of 100 rpm. Three parallel samples were set for each group. MA powder was added to phosphate-buffered saline (PBS) solution to prepare a liquid equivalent to 0.6 mg·ml^−1^ of MA. The liquid (1 ml) was taken and placed in a dialysis bag, which was then immersed in 10 ml of PBS (pH 7.4) solution. Samples of 1 ml were taken at time points (0.33, 0.67, 1, 2, 4, 6, 8, 10, 12, 24, 48, and 72 h), and 1 ml of the release medium at the same temperature was added. The released samples were injected for analysis under the set chromatographic conditions. The peak areas were recorded, and the cumulative release rate *Q* was calculated. In the same way, a PBS solution of MA + SeNPs test samples with the same concentration was used as a control for the in vitro release test. The peak areas were recorded, and the cumulative dissolution rate was calculated.

### Drug stability investigation

Se@CSNPs, Se@CS@MANPs, and Se@CS@MA@HANPs were stored at 4 °C for 30 d. The particle size and zeta potential of the nanoparticles were measured every 5 d using a laser particle size analyzer to evaluate their stability.

### Cellular uptake

Coumarin 6 (C6) was used as a probe to investigate the uptake of nanoparticles by cells. For RAW264.7 cells, 3 groups were set up: the free C6 group, the Se@CS@C6NPs group, and the Se@CS@C6@HANPs group, with 3 replicate wells in each group. Cell suspension (500 μl) with a density of 1 × 10^4^ cells per well was inoculated into 24-well plates containing coverslips and cultured in an incubator for 24 h. After aspirating and discarding the old culture medium, 3 types of culture media containing C6 were added. After incubation in the incubator for 2 h, the old culture medium was aspirated and discarded, and the cells were washed 3 times with cold PBS. Then, the cells were fixed with 4% paraformaldehyde for 20 min and washed 3 times with cold PBS again. The coverslips were taken out and mounted on slides with anti-fluorescence quenching mounting medium containing DAPI (4′,6-diamidino-2-phenylindole) for 10 min. Subsequently, each slide was placed under a laser confocal scanning microscope to observe the cell uptake of each C6 preparation group.

For LPS-induced M1 macrophages, the Se@CS@C6NPs group and the Se@CS@C6@HANPs group were set up, and the cell uptake experiment was carried out in the same way.

### Cell culture and treatments

The MLE-12 cell line was cultured in phenol red-free high-glucose DMEM with 10% FBS, 100 U/ml penicillin, and 0.1 mg/ml streptomycin. After a 24-h incubation at 37 °C with 5% CO₂, the cells were divided into 5 experimental groups (6 duplicates each): H1N1-infected cells [multiplicity of infection (MOI) = 5] were treated with MA + SeNPs (1 μg/ml Se@CS@MA@HANPs), MANPs (1 μg/ml CS@MA@HANPs), and SeNPs (1 μg/ml Se@CS@HANPs). To induce infection, all groups except Con were exposed to H1N1 at a MOI of 5, while Con and Vir were given blank medium. After treatment, cells were grown for 48 h at 37 °C and 5% CO₂, with daily monitoring of cytopathic effects (CPEs). Cell viability was determined using the Cell Counting Kit-8 (CCK-8) test. The levels of IL-6, TNF-α, IL-1β, and IL-10 in the supernatants of cells from each experimental group were detected by ELISA.

### ROS detection

Intracellular reactive oxygen species (ROS) levels were quantified using the fluorescent probe 2′,7′-dichlorodihydrofluorescein diacetate (DCFH-DA). MLE-12 cells were seeded in 96-well plates at a density of 1 × 10^4^ cells per well and allowed to adhere for 24 h. After the incubation period, cells were treated with curcumin or dimethyl sulfoxide (vehicle control) for 48 h. As a positive control, tert-butyl hydroperoxide (tBHP) was administered 4 h prior to staining. For the detection procedure, the culture medium was first removed, and cells were washed with 100 μl of 1× assay buffer per well. Following buffer removal, cells were incubated with 100 μl of 10 μM DCFH-DA solution at 37 °C for 45 min under light-protected conditions. After removing the dye, fluorescence intensity was immediately measured using a microplate reader with excitation/emission wavelengths set at 485/535 nm. Quantitative image analysis was performed using ImageJ software, with fluorescence intensities normalized to the control group values.

### Detection of H1N1 NP protein in cells

The expression of the H1N1 viral NP protein in cells from each experimental group was detected and colocalized using immunofluorescence and confocal laser microscopy to assess the changes in viral particles within host cells after drug treatment.

### Animal experiments

Specific pathogen-free (SPF) KM mice were obtained from Hunan Silek Jingda Laboratory Animal Co. Ltd. and acclimatized for 7 d under standardized conditions. Then, mice were randomly divided into 5 groups according to their body weight: the Normal group (Nor), the Model group (Mod), MA + SeNPs group (treated by Se@CS@MA@HANPs), MANPs group (treated by CS@MA@HANPs), and SeNPs (treated by Se@CS@HANPs group), with 8 mice in each group. On the first day, all mice except those in the Nor group were intranasally infected with ×10 minimum lethal dose (MLD_50_) of H1N1 viruses in a 50-μl volume. Mice in the treatment group were administered the corresponding nanoparticles intranasally at a dose of 5 ml/kg daily, while the mice in the Nor and Mod groups received equivalent volumes of deionized water daily. The body weight and survival rate of the mice in each group were monitored daily. On day 14 post-infection, mice were euthanized by cervical dislocation, and lungs were dissected out.

### Lung index and hematoxylin and eosin staining

Under sterile conditions, the lung tissues were rapidly excised, washed with PBS to remove residual surface blood, and then placed on sterile filter paper to remove excess moisture. The weight of the lung tissue was measured, and the lung index was calculated (lung tissue weight/body weight × 100%). The degree of pulmonary edema in the lungs of mice in each group was analyzed. Besides, lung tissues were fixed. The samples were dehydrated in ethanol, embedded with paraffin, and sliced. Then, the lung tissues were deparaffinized in xylene for 10 min and stained with hematoxylin and eosin (H&E) for histopathological examination of lung inflammation.

### Lung tissue cytokine analysis

Lung tissue (100 mg) from each experimental group of mice was taken, homogenized in a homogenizer after adding PBS (lung tissue:PBS = 1:9), and then centrifuged at 12,000 rpm for 10 min. The supernatant was collected, and the levels of IL-18, IL-1β, TNF-α, IL-6, SOD, and GPx in the lung tissue homogenate were detected using the ELISA method.

### Immunohistochemical detection

Mouse lung tissues were taken and fixed with 4% paraformaldehyde, then dehydrated with gradient ethanol, cleared with xylene, and embedded in paraffin. The tissue was cut into sections 4 to 5 μm thick. For antigen retrieval, the sections were placed in 10 mM sodium citrate buffer (pH 6.0) and heated in a microwave for 8 to 15 min. After blocking with 3% bovine serum albumin (BSA)–PBS at room temperature for 30 min, NP antibody (diluted at 1:1,000) was added and incubated overnight at 4 °C. Following PBS washes, goat anti-rabbit IgG was added and incubated at room temperature for 1 h. The sections were then treated with streptavidin–horseradish peroxidase (SA-HRP) complex and developed with DAB (3,3′-diaminobenzidine). The sections were counterstained with hematoxylin and mounted with neutral gum. Finally, the sections were observed under an optical microscope, and the expression of NP in the mouse lung tissue was assessed based on the staining results.

### Quantitative RT-PCR

Approximately 15 to 20 mg of pulmonary tissue were homogenized in 300 μl of lysis buffer using a high-speed tissue disruptor to generate a uniform cell suspension. The extracted total RNA was quantified via ultramicrospectrophotometry and subsequently reverse-transcribed into complementary DNA (cDNA). For quantitative analysis, SYBR Green-based real-time polymerase chain reaction (RT-PCR) (Qiagen, Hilden, Germany) was employed to assess mRNA expression. The primer sequences targeted the murine housekeeping gene PPIA and the influenza viral M segment, designed as follows: viral M gene: forward: 5′-CTTCTAACCGAGGTCGAAAC-3′, reverse: 5′-CGTCTACGCTGCAGTCCTC-3′; PPIA (internal control): forward: 5′-CGCTTGCTGCAGCCATGGTC-3′, reverse: 5′-CAGCTCGAAGGAGACGCGGC-3′. The relative quantification of influenza virus M gene copies was computed using the 2^−ΔΔCT^ method for comparative threshold cycle analysis.

### Effect of Se-MANPs on PANoptosis of MLE-12 cell

The MLE-12 cell line was maintained in phenol red-free high-glucose DMEM supplemented with 10% FBS, 100 U/ml penicillin, and 0.1 mg/ml streptomycin. Following a 24-h incubation at 37 °C under 5% CO₂, the cells were allocated into 3 experimental groups (6 replicates per group): Normal (Nor): untreated cells, Virus (Vir): H1N1-infected cells (MOI = 5), and MA + SeNPs (1 μg/ml Se@CS@MA@HANPs). All groups except Con were exposed to H1N1 at an MOI of 5 to induce infection, while Con and Vir received blank medium. Post-treatment, cells were cultured for 48 h (37 °C, 5% CO₂).

Cells were lysed using radioimmunoprecipitation assay lysis buffer to extract total protein, and the protein concentration was determined using the BCA kit or other methods. Based on the protein concentration, an appropriate amount of protein sample was mixed with loading buffer, boiled to denature the proteins, and then subjected to sodium dodecyl sulfate–polyacrylamide gel electrophoresis (SDS-PAGE) electrophoresis. The gel concentration was selected according to the molecular weight of the target proteins to achieve separation. The separated proteins were transferred from the gel to a polyvinylidene difluoride membrane using the wet transfer method, with appropriate voltage and time settings to ensure complete transfer. The membrane was blocked with 5% skimmed milk powder at room temperature for 1 h. Specific primary antibodies against ZBP1, RIPK3, MLKL, GSDMD, caspase-3, and caspase-7 (diluted at a ratio of 1:1,000) were added and incubated overnight at 4 °C. The membrane was then washed 3 to 5 times with tris-buffered saline with Tween 20 (TBST) for 5 to 10 min each time. HRP-conjugated secondary antibodies matching the primary antibodies were added and incubated at room temperature for 1 h. The membrane was washed again 3 to 5 times with TBST to remove unbound secondary antibodies. Finally, enhanced chemiluminescence substrate was added, and the membrane was imaged using a chemiluminescent imaging system in a dark room to analyze the expression levels of the target proteins.

### Evaluation of the biosafety of MA + SeNPs in mice

Mice were divided into 2 groups (*n* = 10 per group): the PBS group and the MA + SeNPs group. Both groups received daily intranasal administration of PBS or MA + SeNPs for 14 consecutive days. At the end of the experiment, all mice were humanely euthanized, and major organs (heart, liver, spleen, lungs, kidneys, and brain) were collected for histopathological examination using H&E staining.

### Statistical analysis

The data were expressed as mean ± standard error of mean (SEM). Statistical analysis was conducted using one-way analysis of variance (ANOVA) with Duncan’s multiple comparison test on SPSS 27.0 software. All difference with *P* < 0.05 were considered to indicate significance.

## Results

### Characterization of Se@CS@MA@HANPs

SEM and TEM analyses of the prepared nanoparticles revealed that Se@CS@MA@HANPs exhibited a quasi-spherical structure with good dispersibility (Fig. 1B). Based on SEM and TEM results, energy-dispersive x-ray spectroscopy (EDS) was further employed to analyze the elemental composition and distribution of Se in Se@CS@MA@HANPs, with the results shown in Fig. 1C. In the prepared nanoparticles, selenium accounted for 45.31%, carbon for 6.48%, and oxygen for 21%. The signal of selenium was primarily concentrated within the signal regions of carbon and oxygen, showing a highly overlapping distribution with these elements. This suggests that selenium is likely uniformly encapsulated in the form of nanoparticles within an organic matrix rich in carbon and oxygen, indicating a good binding or encapsulation relationship between selenium and the organic components.

Additionally, particle size analysis revealed that on the basis of the spherical structure of Se@CSNPs (with a particle size of 66.32 ± 0.68 nm), the particle size of Se@CS@MANPs increased to 113.49 ± 1.40 nm upon DL. After modification with HA, the particle size of Se@CS@MA@HANPs further increased to 208.80 ± 6.46 nm, with a zeta potential of 12.85 ± 2.46 mV, as shown in Fig. 1D. Upon testing, the EE and DL of Se@CS@HANPs for MA are 93.45% and 60.87%, respectively.

FTIR was further employed to characterize different nanoselenium particles as well as MA and HA, as shown in Fig. 1E. The broad peak at 3,400 cm^−1^ corresponds to the characteristic absorption of O–H stretching vibrations, indicating the presence of intramolecular or intermolecular hydrogen bonds. The peaks near 2,920 and 1,612 cm^−1^ in Se@CSNPs (a), Se@CS@MANPs (b), and Se@CS@MA@HANPs (c) are attributed to the C–H stretching vibrations of methyl and methylene groups from residual sugar moieties and the carbonyl C=O stretching vibrations, respectively. The absorption peak at 1,384 cm^−1^ is typically associated with the symmetric deformation vibration of -CH₃ (methyl) groups. The C–O stretching vibration absorption peaks are observed at 1,300 and 1,070 cm^−1^, while the peak near 897 cm^−1^ corresponds to the characteristic absorption of the β-glycosidic bond in chitosan. All these absorption peaks exhibited varying degrees of redshift upon the addition of MA and HA. The peak near 1,450 cm^−1^ in Se@CS@MANPs (b), Se@CS@MA@HANPs (c), and MA (d) is characteristic of aromatic rings, confirming the successful dispersion of MA on the surface of nanoselenium. The peak near 1,662 cm^−1^ in Se@CS@MA@HANPs (c) and HA (e) corresponds to the C=O stretching vibration in secondary amides, indicating the successful encapsulation of nanoselenium particles by HA.

The intensity and sharpness of XRD peaks can reflect the crystalline nature of selenium to some extent. As shown in Fig. 1F, the XRD pattern of Se@CSNPs (a) exhibits 2 sharp characteristic peaks at 2θ = 24° and 30°, along with broad diffraction peaks in the range of 20° to 40°, indicating that SeNPs exist in an amorphous form. HA (e) shows broad diffraction peaks in the range of 10° to 40°, indicating its amorphous nature. MA (d) has a characteristic peak at 42°. The diffraction patterns of Se@CS@MANPs (b) and Se@CS@MA@HANPs (c) not only retain the characteristic peaks of Se@CS but also preserve the 42° peak of MA, suggesting that the combination of Se@CSNPs with MA maintains their respective structural regularities.

### The stability of the drug

**Fig. 2.**
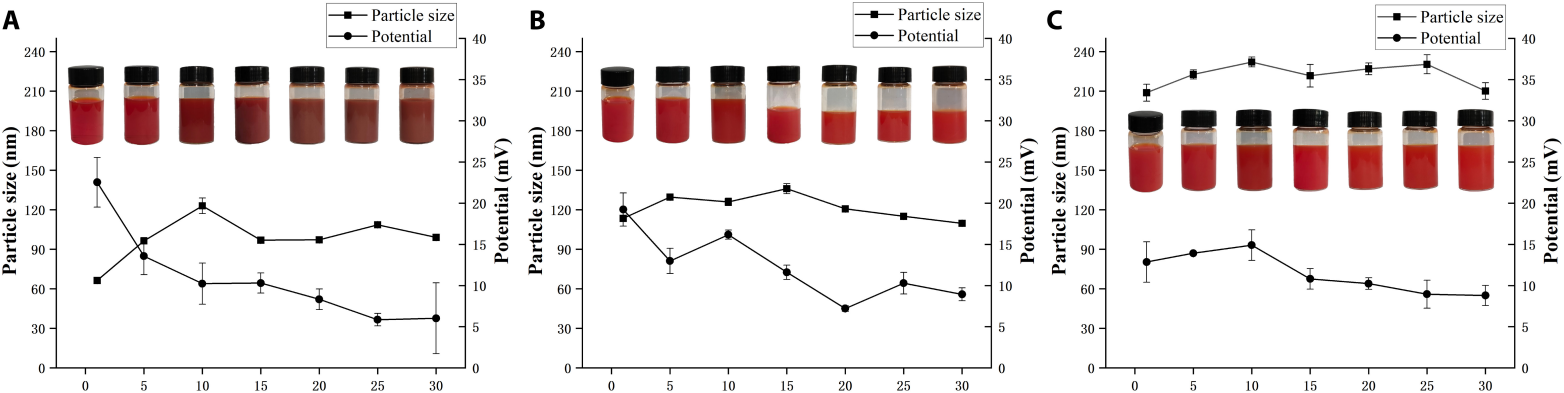
Particle size and zeta potential of nanoparticles. (A) Se@CSNPs. (B) Se@CS@MANPs. (C) Se@CS@MA@HANPs.

Figure 2 illustrates the changes in particle size and zeta potential of nanoselenium over a period of 30 d. As shown in the figure, freshly prepared Se@CSNPs (Fig. 2A), Se@CS@MANPs (Fig. 2B), and Se@CS@MA@HANPs (Fig. 2C) all exhibited an orange-red color and were clear and transparent. Subsequently, Se@CS gradually turned dark red, while the other 2 showed no significant color change. It is speculated that Se@CS might have been oxidized, leading to the gradual transformation of nanoselenium into elemental selenium. In contrast, the other 2 formulations remained relatively stable after DL. From the particle size changes depicted in the figure, we can observe that the particle size of Se@CSNPs increased rapidly from 66 ± 0.68 nm within the first 10 d and eventually stabilized around 99 ± 2.2 nm. The particle sizes of the other 2 formulations changed little, which also indicates that the stability of the nanoparticles was enhanced after DL. The zeta potential values shown in the figure for Se@CSNPs (Fig. 2A), Se@CS@MANPs (Fig. 2B), and Se@CS@MA@HANPs (Fig. 2C) were 22.53 ± 3.01 mV, 19.23 ± 2.01 mV, and 12.85 ± 2.46 mV, respectively. All 3 exhibited a downward trend over the 30-d period, eventually stabilizing around 8 mV. The zeta potential of Se@CS decreased the most, a result consistent with the particle size observations. In summary, we can conclude that the stability of Se@CS was significantly improved after DL.

### The drug release test result

**Fig. 3:**
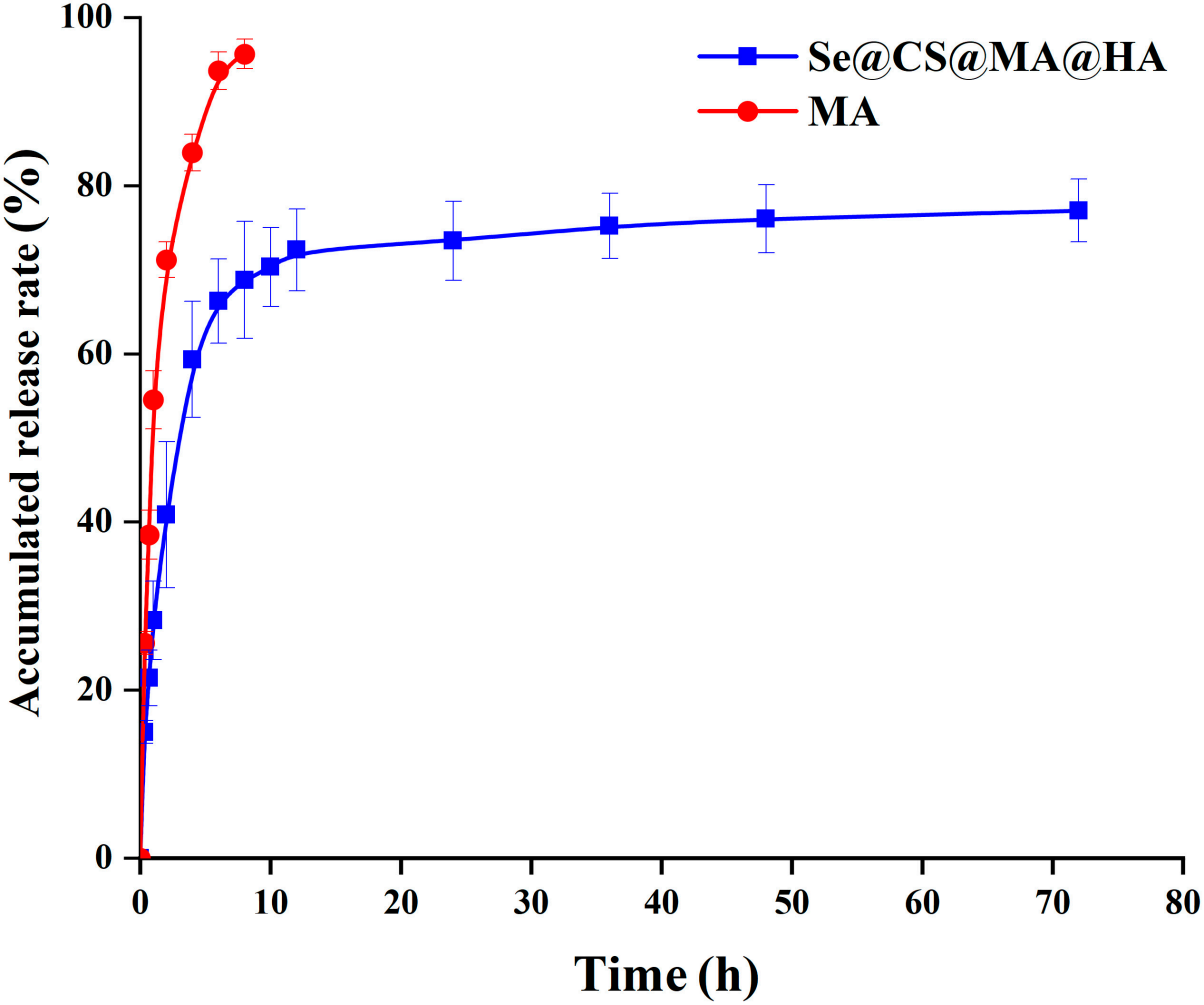
In vitro release of MA from nanoparticles in pH 7.4 at 37 °C temperature.

Figure 3 shows the cumulative release curves of free MA and Se@CS@MA@HANPs in PBS at 37 °C. It could be seen that free MA was almost completely released within 4 h. In contrast, the release of MA from Se@CS@MA@HANPs was relatively rapid in the first 4 h and then slowed down. The cumulative release of MA from Se@CS@MA@HANPs reached 77.02% ± 3.74% within 72 h. Fitting the cumulative release of MA from Se@CS@MA@HANPs (*Q*) revealed that the release of MA from Se@CS@MA@HANPs followed a first-order kinetic equation. The fitting equation is *Q* = 73.58 (1 − *e* − 0.44*t*), with *R*^2^ = 0.9901.

### The uptake of nanoparticles by cells

**Fig. 4.**
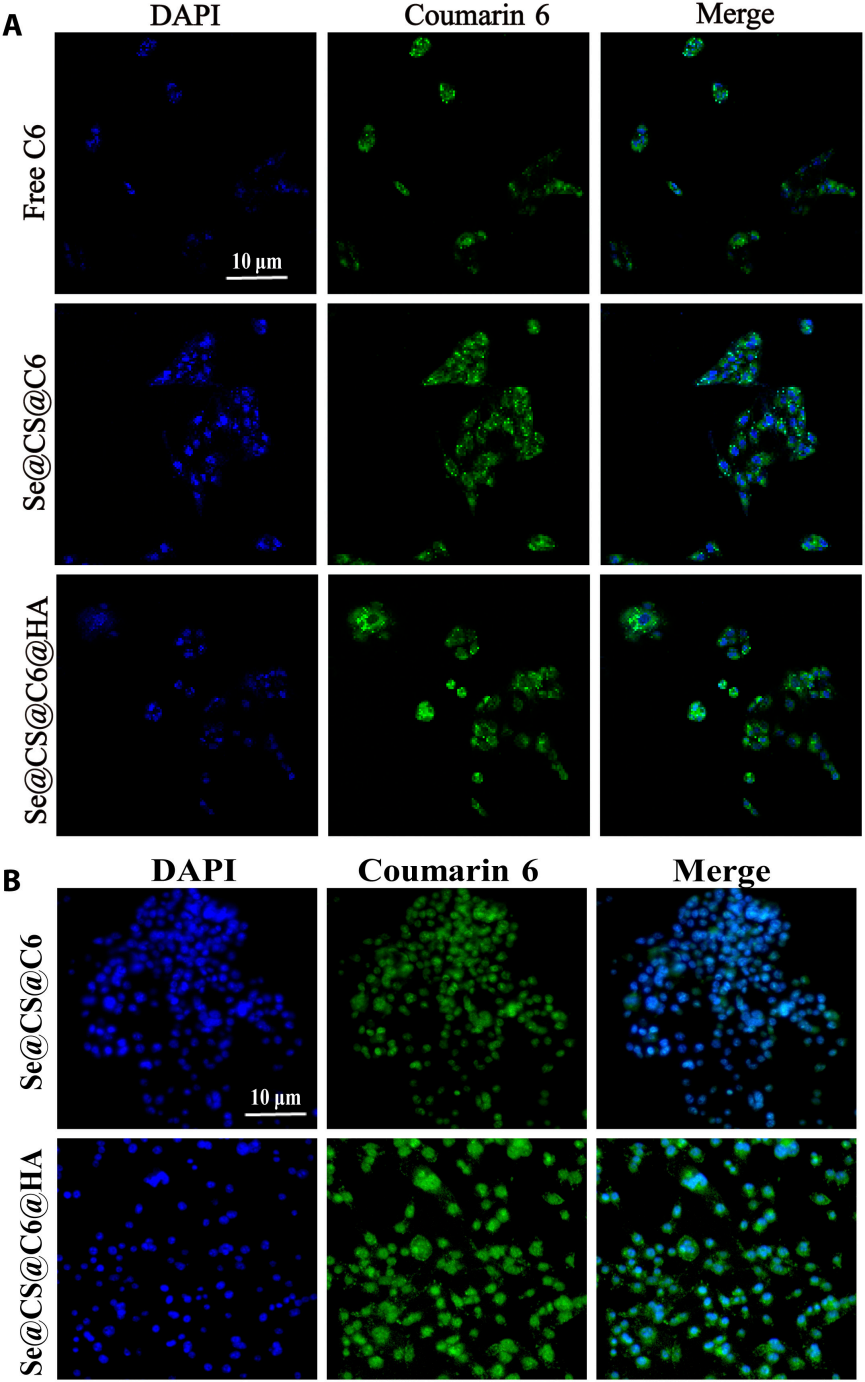
Cellular uptake of nanoparticles encapsulating C6. (A) Cellular uptake of C6 by RAW264.7 cells. (B) Cellular uptake of C6 by M1 macrophages.

In this study, C6 was used as a model probe to investigate the uptake of the prepared nanoparticles by RAW264.7 cells. As shown in Fig. 4A, RAW264.7 cells exhibited weak green fluorescence in the cytoplasm, indicating that the uptake of free C6 by cells was minimal. Compared with cells treated with free C6, cells treated with Se@CS@C6NPs and Se@CS@C6@HANPs showed significantly enhanced green fluorescence, with the strongest fluorescence observed in the Se@CS@C6@HANPs group. This suggests that the prepared nanoparticles possess good characteristics for transmembrane transport across cell membranes. To further investigate the specific affinity of Se@CS@HANPs for inflammatory cells, this study used LPS-induced M1-type macrophages differentiated from RAW264.7 cells as a model to observe the uptake of Se@CS@C6@HANPs and Se@CS@C6NPs by these cells within 2 h. The results showed that M1 macrophages treated with Se@CS@C6@HANPs exhibited significantly higher fluorescence intensity compared to those treated with Se@CS@C6NPs, indicating that nanoparticles modified with HA have better affinity for inflammatory cells, as shown in Fig. 4B.

### In vitro antiviral and anti-inflammatory effects of nanoparticles

**Fig. 5.**
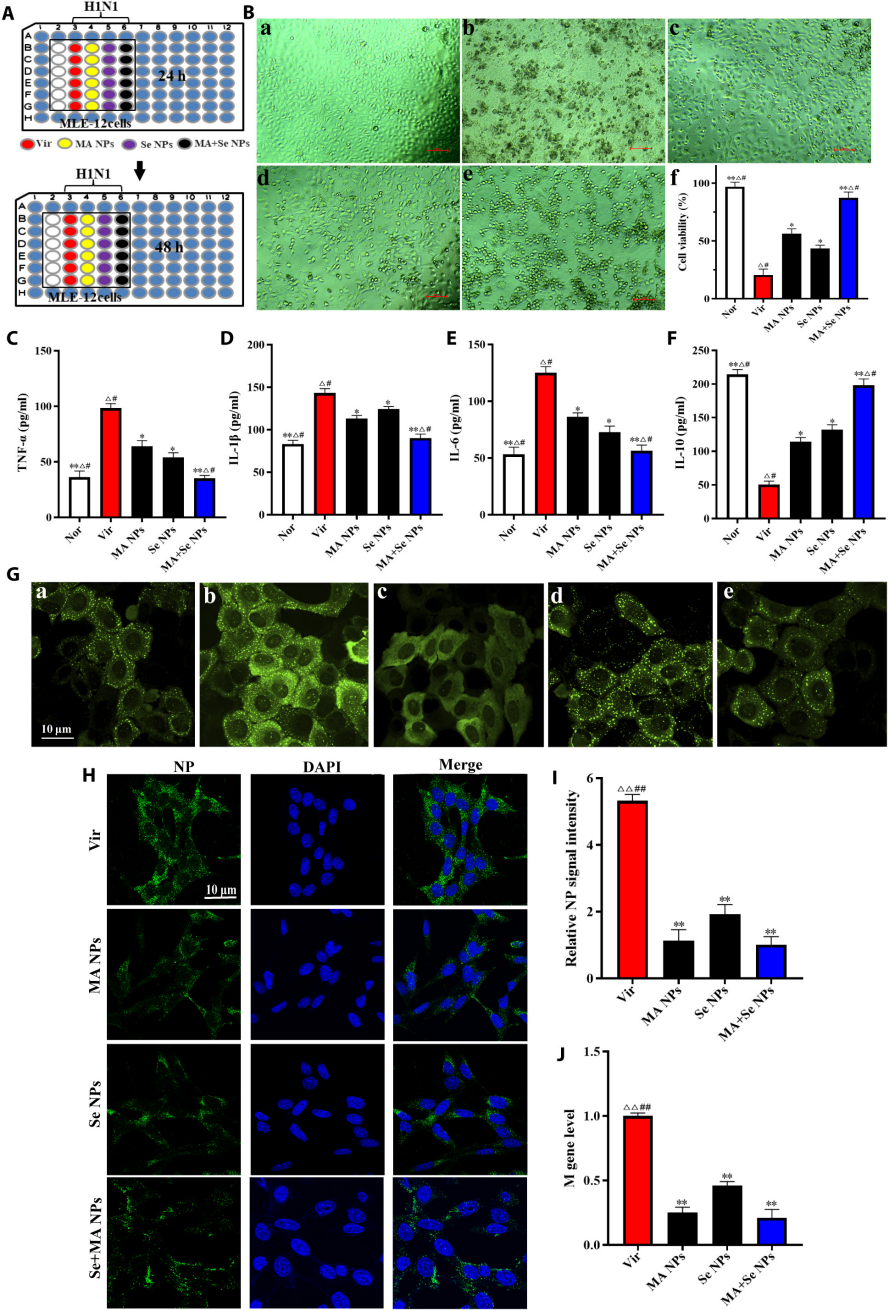
In vitro antiviral and anti-inflammatory effects of MA + SeNPs. (A) Experimental grouping and drug treatment timeline. (B) CPE and cell viability. (C to E) Levels of inflammatory cytokines (TNF-α, IL-1β, and IL-6). (F) Levels of anti-inflammatory cytokine (IL-10). (G) fluorescent probe detection of ROS in MLE-12 cells. (H) Immunofluorescence detection of influenza virus NP protein in MLE-12 Cells. (I) NP protein signal intensity. (J) M gene level of influenza virus detected by QRT-PCR. (a) Nor group. (b) Vir group. (c) MANPs group. (d) SeNPs group. (e) MA + SeNPs group. The results represent the mean ± SD of 3 independent experiments. *P < 0.05, **P < 0.01 versus the Vir group. ^△^*P* < 0.05, ^△△^*P* < 0.01 versus the MANPs group. ^#^*P* < 0.05, ^##^*P* < 0.01 versus the MA + SeNPs group.

To investigate the synergistic advantages of Se and MA combination therapy in antiviral and anti-inflammatory efficacy, MLE-12 cells infected with H1N1 for 24 h were treated with SeNPs, MANPs, or inflammation-responsive nanoparticles coloaded with Se and MA (MA + SeNPs) for 48 h (Fig. 5A). The results showed that SeNPs, MANPs, and MA + SeNPs all alleviated CPEs to varying degrees and inhibited H1N1-induced cell death. Notably, MA + SeNPs demonstrated significantly superior effects in mitigating CPE and enhancing cell viability compared to Se or MA alone (Fig. 5B).

Further analysis of pro- and anti-inflammatory cytokines in host cells revealed that H1N1 infection triggered a substantial increase in TNF-α, IL-1β, and IL-6 levels, while the anti-inflammatory cytokine IL-10 was reduced. After treatment with the 3 nanoparticles, TNF-α, IL-1β, and IL-6 levels in MLE-12 cells decreased significantly, whereas IL-10 levels increased. Importantly, MA + SeNPs exhibited markedly stronger reversal effects on these cytokines than Se or MA alone (Fig. 5C to F). These findings indicate that co-delivery of Se and MA exerts synergistic anti-inflammatory effects.

Studies have shown that ROS can activate signaling pathways such as NF-κB and the NLRP3 inflammasome, promoting the expression and release of pro-inflammatory cytokines (e.g., TNF-α, IL-1β, and IL-6). Excessive ROS also induces oxidative stress, exacerbating inflammatory responses. Influenza virus infection further amplifies ROS production in host cells, triggering inflammation [20]. In our study, H1N1-infected MLE-12 cells exhibited higher ROS levels than uninfected cells (Fig. 5G). Treatment with SeNPs, MANPs, or MA + SeNPs reduced intracellular ROS, with SeNPs and MA + SeNPs showing the most pronounced effects, underscoring the critical role of Se in scavenging virus-induced ROS.

Additionally, the combination of Se and MA exhibited a synergistic inhibitory effect against H1N1 virus. Immunofluorescence and quantitative RT-PCR (qRT-PCR) analyses of viral NP protein and M gene expression revealed that all 3 nanoparticles suppressed H1N1 viral NP protein and M gene to varying degrees, with MA + SeNPs demonstrating the most potent inhibition (Fig. 5H to J).

### In vivo antiviral and anti-inflammatory effects of nanoparticles

**Fig. 6.**
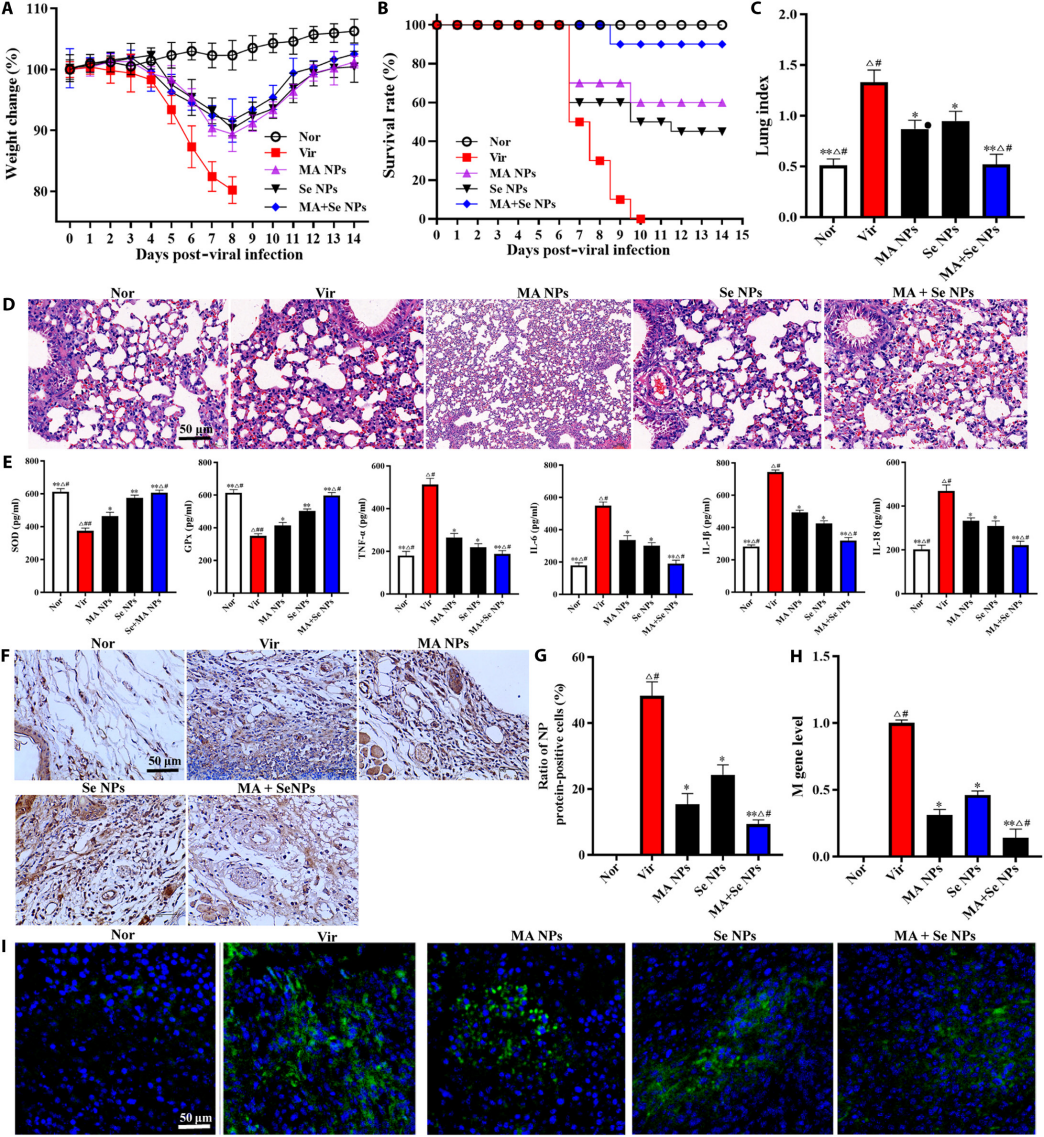
In vivo antiviral and anti-inflammatory effects of MA + SeNPs. (A) Comparison of body weight changes among different experimental groups of mice. (B) Effect of MA + SeNPs on the survival rate of H1N1-infected mice. (C) Lung index. (D) H&E staining of lung tissue sections. The histopathological changes are indicated by arrows. (E) Levels of SOD, GPx, TNF-α, IL-6, IL-1β, and IL-18 in mouse lungs. (F) Immunohistochemical detection of NP protein in mouse lung tissues. (G) Ratio of NP protein-positive cells. (H) M gene level of influenza virus detected by QRT-PCR. (I) Fluorescent probe detection of ROS in mouse lungs. The results represent the mean ± SD of 6 independent experiments. *P < 0.05, **P < 0.01 versus the Vir group. ^△^*P* < 0.05, ^△△^*P* < 0.01 versus the MANPs group. ^#^*P* < 0.05, ^##^*P* < 0.01 versus the MA + SeNPs group.

To evaluate the protective efficacy of MA + SeNPs against H1N1 infection in mice, intranasal delivery was employed. Mice infected with H1N1 were administered MANPs, SeNPs, or MA + SeNPs, followed by monitoring of survival rates and body weight changes over 14 d. As illustrated in Fig. 6A, by day 8 post-infection (dpi), the H1N1-challenged group exhibited a 20.12% reduction in body weight. In contrast, the MANPs, SeNPs, and MA + SeNPs treatment groups demonstrated significantly lower weight losses of 11.6%, 9.66%, and 8.37%, respectively. Similarly, survival rates varied markedly among groups (Fig. 6B). While only 12.5% of the virus-infected control mice survived, the MANPs, SeNPs, and MA + SeNPs groups showed survival rates of 45%, 60%, and 90%, respectively. Notably, mice treated with 3 types of NPs displayed a modest reduction in weight loss between 2 and 8 dpi compared to the H1N1-infected group. By 8 dpi, surviving animals in the nanoparticle-treated groups began regaining weight, whereas the untreated infected mice showed minimal recovery. Control mice remained unaffected, displaying no clinical symptoms, weight decline, or mortality. These findings indicate that nanoparticle intervention can mitigate H1N1-induced weight loss and enhance survival in infected mice. At the same time, these 3 types of nanoparticles can also significantly reduce the lung index of mice (Fig. 6C) and can improve the lung injury in mice caused by the H1N1virus to a certain extent (Fig. 6D). It is worth noting that the combined use of MA and Se has a significantly better improvement effect on the abovementioned indicators than when they are used alone.

What is more, the combined use of MA and Se has an important impact on the balanced regulation of immunity and inflammation in mice with viral pneumonia. As shown in Fig. 6E, after being infected with the H1N1 virus, a large amount of inflammatory factors IL-1β, IL-18, IL-6, and TNF-α were produced in the lung tissues of mice, while the antioxidant factors SOD and GPx were significantly reduced, exhibiting oxidative inflammatory damage in lung tissue. However, after the mice were treated with MA and Se, the levels of IL-1β, IL-18, IL-6, and TNF-α in the lung tissues of the mice were effectively controlled, and the levels of SOD and GPx were effectively restored. Moreover, the reversing effect of the combined use of MA and Se on these cytokines was significantly better than that of using a MA and Se alone.

Further detection by immunohistochemistry and qRT-PCR revealed abundant NP protein-positive cells (Fig. 6F and G) and viral M gene expression (Fig. 6H) in the lung tissues of H1N1-infected mice. Following treatment with the 3 types of nanoparticles, both NP protein-positive cells and viral M gene levels were significantly reduced. Among them, MA + SeNPs exhibited the strongest antiviral efficacy, followed by MANPs, while SeNPs showed the weakest effect. These results fully demonstrate that the combination of MA and Se exerts a synergistic antiviral effect.

To investigate the oxidative stress status in the lung tissues of viral pneumonia mice, we detected ROS levels in mouse lungs. As shown in Fig. 6I, H1N1 infection induced massive ROS production in lung tissues. Studies indicate that excessive ROS can damage pulmonary cells (including epithelial and endothelial cells), leading to cell membrane lipid peroxidation, protein oxidative modification, and DNA damage, ultimately causing cellular dysfunction or even death [21]. This process further exacerbates pulmonary inflammatory responses by promoting the release of inflammatory cytokines, creating a vicious cycle of inflammation. Consequently, lung tissue structure and function become impaired, manifesting as pathological changes such as increased alveolar–capillary permeability, pulmonary edema, and fibrosis—severely compromising normal respiratory function [22,23]. Notably, nanodrug treatment significantly reduced ROS levels in lung tissues, particularly in the SeNPs and MA + SeNPs groups. These results clearly demonstrate the critical role of Se in scavenging excess ROS induced by viral infection.

The above findings indicate that the co-delivery of MA and Se achieves coordinated control of viral load and inflammation in the lungs of viral pneumonia mice while also effectively mitigating influenza virus-induced excessive oxidative stress in lung tissues.

### MA + SeNPs inhibits influenza virus-induced PANoptosis in MLE-12 cells

**Fig. 7.**
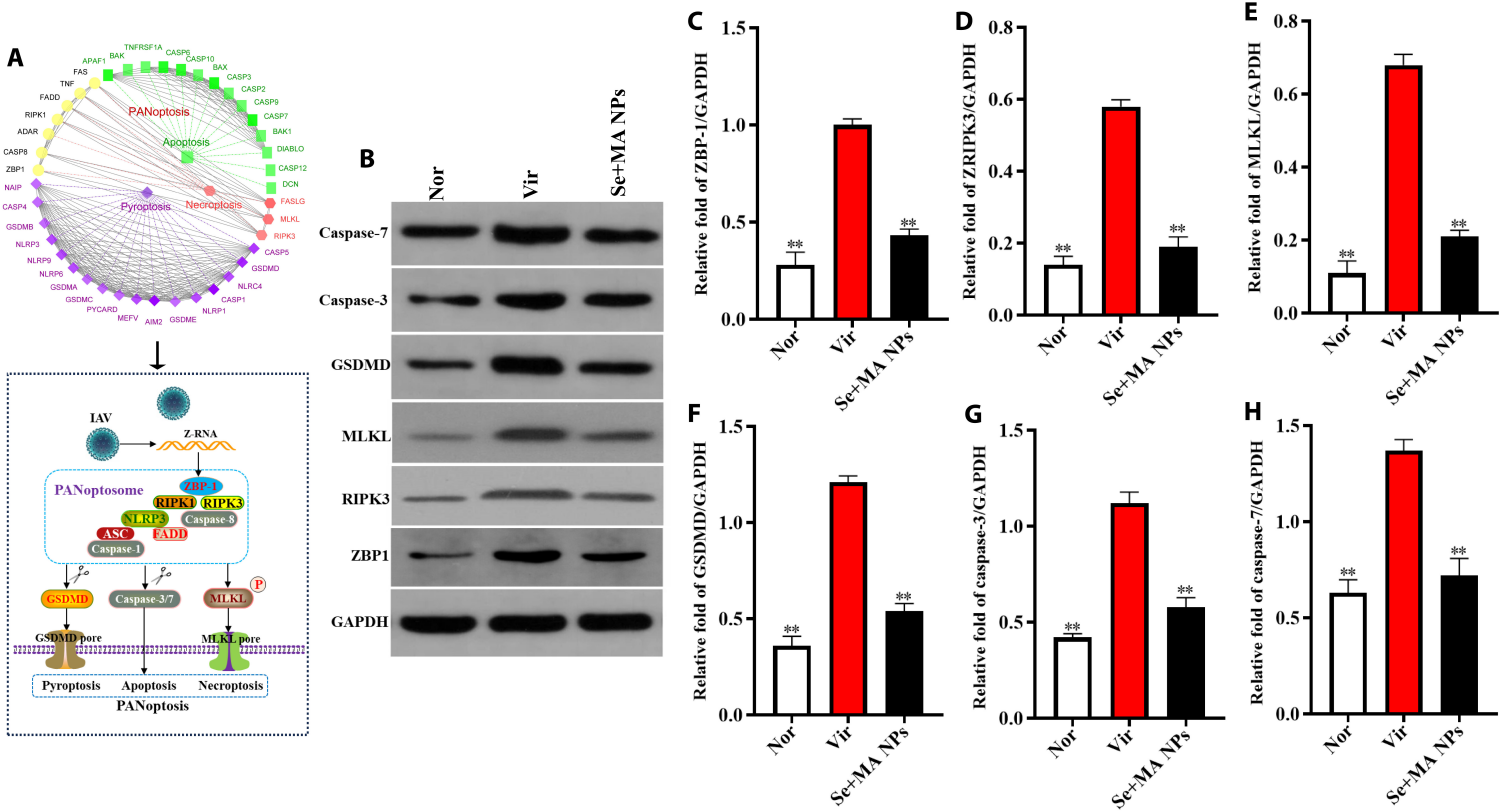
Effect of MA + SeNPs on H1N1-induced PANoptosis in MLE-12 cell*s*. (A) Mechanism and key effector proteins of PANoptosis. (B) Western blot detection of PANoptosis-associated key proteins. (C to H) Expression of ZBP1, RIPK3, MLKL, GSDMD, caspase-3, and caspase-7. The results represent the mean ± SD of 3 independent experiments. *P < 0.05, **P < 0.01 versus the Vir group.

Influenza virus infection exacerbates lung injury through the induction of PANoptosis, a process involving the activation of the host protein Z-DNA binding protein 1 (ZBP1) by viral RNA [24]. This activation triggers the rupture of the cell membrane via the RIPK3/MLKL pathway and the amplification of inflammation in a caspase-8-dependent manner. The death of alveolar epithelial and vascular endothelial cells leads to the collapse of barrier functions, resulting in the massive release of inflammatory factors (such as IL-6 and TNF-α) and damage-associated molecular patterns (DAMPs), which in turn trigger cytokine storms and immune cell infiltration [25]. PANoptosis acts in concert with pyroptosis [mediated by gasdermin D (GSDMD)] and apoptosis, further aggravating alveolar structural destruction, microthrombus formation, and hypoxia. The excessive inflammation and imbalance in tissue repair ultimately lead to ARDS and multi-organ failure, which are core pathological mechanisms underlying the severity of influenza. Targeted inhibition of ZBP1 or RIPK3 may serve as potential therapeutic strategies. The mechanism of PANoptosis is illustrated in Fig. 7A.

After influenza virus infection, the PANoptosis-related proteins ZBP1, RIPK3, MLKL, GSDMD, caspase-3, and caspase-7 were significantly up-regulated in MLE-12 cells, as shown in Fig. 7B to H. indicating that the H1N1 virus triggers pyroptosis, apoptosis, and necroptosis simultaneously via the ZBP1 pathway, which is defined as PANoptosis. However, after intervention with MA + SeNPs, these proteins were significantly down-regulated, demonstrating the inhibition of PANoptosis. This may be an important mechanism by which MA + SeNPs suppress the cytokine storm in mouse lung tissues.

### The biosafety evaluation of MA + SeNPs

**Fig. 8.**
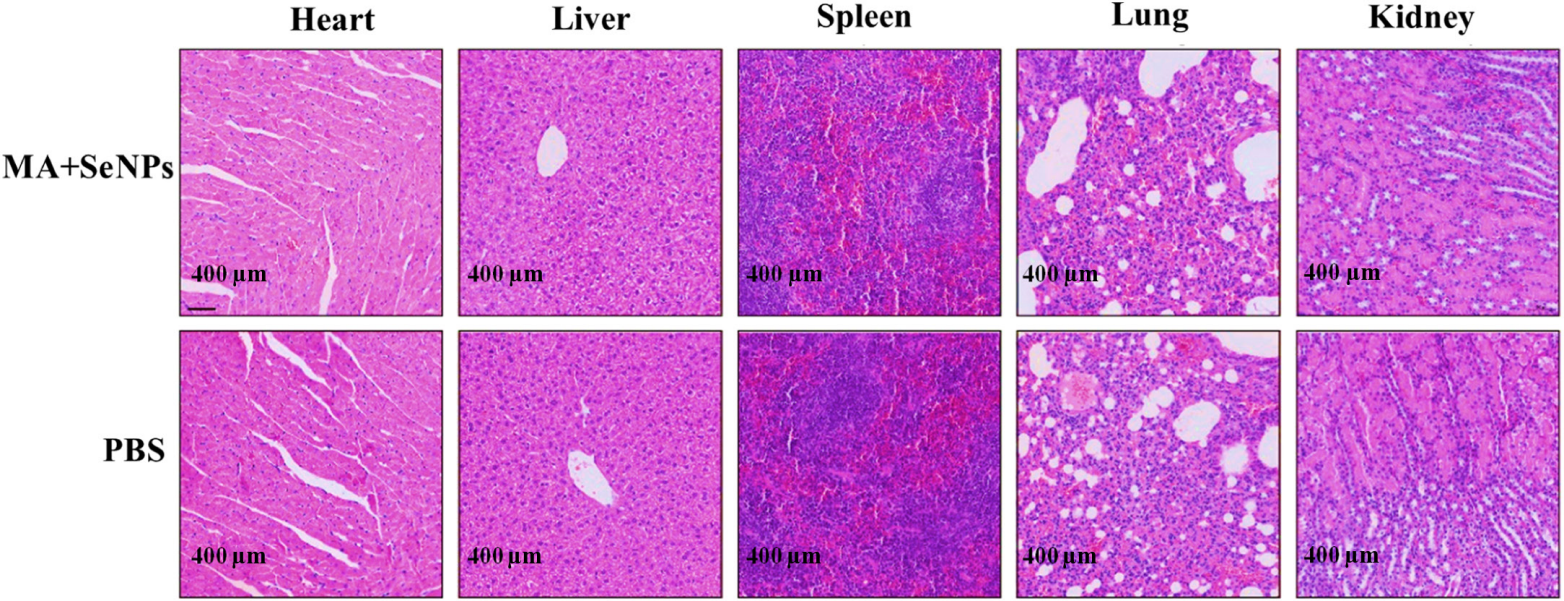
The safety evaluation of MA + SeNPs treatment in vivo*.*

After 14 consecutive days of intranasal administration, we found that MA + SeNPs did not exhibit significant toxic effects on the heart, liver, spleen, lungs, or kidneys of mice, as shown in Fig. 8. Histopathological examination of these vital organs revealed no observable cellular damage, inflammatory responses, or other abnormal pathological changes. These results demonstrate that MA + SeNPs possess excellent biocompatibility and low toxicity risks in vivo, indicating favorable biosafety profiles.

## Discussion

Virus-induced lung injury typically results from the dual effects of rapid viral replication and excessive host immune responses. While antiviral drugs alone can reduce viral load, they fail to promptly suppress inflammatory spread; conversely, anti-inflammatory therapy alone may lead to disease relapse and exacerbation due to incomplete viral clearance. Dual intervention, by simultaneously inhibiting viral replication and modulating inflammatory pathways, not only disrupts pathogen transmission but also mitigates tissue damage, thereby breaking the “virus-inflammation” positive feedback loop. This approach significantly reduces the risk of acute lung injury progressing to ARDS and improves patient survival rates. The synergistic mechanism of antiviral and anti-inflammatory actions provides a critical theoretical foundation for this study. MA, an antiviral compound, was combined with anti-inflammatory SeNPs to achieve multi-target antiviral effects, offering a novel nanodelivery strategy for the treatment of viral pneumonia.

In viral pneumonia, ROS exacerbate lung injury through a dual mechanism. On the one hand, viral replication induces mitochondrial dysfunction and the activation of nicotinamide adenine dinucleotide phosphate (NADPH) oxidase, leading to an excessive production of ROS. This triggers lipid peroxidation, protein inactivation, and DNA damage, directly destroying alveolar epithelial and vascular endothelial cells [26]. On the other hand, ROS amplifies the inflammatory response by activating signaling pathways such as NF-κB and mitogen-activated protein kinases (MAPKs), promoting the release of inflammatory cytokines (such as IL-6 and TNF-α) and forming a “cytokine storm” [27]. Meanwhile, ROS collaborate with cell death pathways such as PANoptosis and pyroptosis. Through oxidative stress, ROS enhance the activity of the ZBP1/RIPK3/MLKL pathway, exacerbating cell membrane rupture and the release of DAMPs, resulting in the collapse of the alveolar–capillary barrier, microthrombus formation, and hypoxia. Excessive ROS can also inhibit the antioxidant system (such as SOD and glutathione), creating a vicious cycle of oxidative stress, ultimately promoting the development of ARDS and multiple organ failure [28]. Regulating ROS levels is an important therapeutic strategy for controlling viral pneumonia.

After infection with the H1N1 virus, we detected a large number of viral particles and ROS in the lung tissues of mice. In addition, inflammatory cytokines such as TNF-α, IL-1β, and IL-6 were highly active, while antioxidant factors such as SOD and GPx were decreased. This is clearly the direct cause of the high mortality rate of mice in the infected group and the severe pulmonary inflammation. After treatment with MA + SeNPs, the survival rate of the mice increased significantly. The viral particles and ROS in the lung tissues were significantly reduced. The lung index and the levels of pulmonary inflammatory cytokines were significantly decreased, and the inflammatory damage to the lung tissues was significantly alleviated. This indicates that MA + SeNPs have successfully achieved the synergistic effect of antiviral and anti-inflammatory pharmacodynamics.

As an important intracellular pattern recognition receptor (PRR), ZBP1 can precisely recognize dangerous signals such as nucleic acids released during influenza virus infection [29]. Once it recognizes the molecular patterns associated with the influenza virus, ZBP1 rapidly activates its own functions, recruits key proteins such as RIPK1/3 and MLKL, and initiates the PANoptosis signaling pathway [30]. This pathway integrates multiple cell death pathways, including necroptosis, apoptosis, and pyroptosis, which prompts cells to undergo intense inflammatory death reactions and release a large number of DAMPs, further exacerbating the pulmonary inflammatory response. ZBP1 plays a crucial role in the lung tissue damage and disease progression caused by influenza virus infection. In our study, we also found that after MLE-12 cells were infected with H1N1, the expression of ZBP1 was significantly increased, which further activated the ZBP1-RIPK3-MLKL-caspase signaling axis. At the same time, it induced apoptosis, necroptosis, and pyroptosis, and triggered PANoptosis, leading to the death of MLE-12 cells and the exacerbation of inflammation. After the cells were intervened with MA + SeNPs, the PANoptosis of MLE-12 cells was inhibited. We speculate that there may be 2 pathways. Firstly, after MA inhibits the virus, the expression of ZBP1 decreases, because the expression of ZBP1 is positively correlated with the number of viruses. The decrease in ZBP1 is equivalent to turning off the core initiating sensor of PANoptosis. On the other hand, the scavenging of ROS by Se is also crucial for inhibiting PANoptosis. ROS can activate ZBP1 through oxidative modification and then trigger necroptosis. ROS can also activate caspase-1 or caspase-11. These cysteine proteases are able to cleave GSDMD to form pore-forming proteins, resulting in the rupture of the cell membrane and triggering pyroptosis [31]. In addition, ROS can activate caspase-8 through oxidative modification and then initiate the apoptosis pathway. The activation of caspase-8 will further amplify the inflammatory response and promote the release of inflammatory factors [32].

PANoptosis is a form of cell death that integrates apoptosis, necroptosis, and pyroptosis, and its mechanism involves the cross-activation of multiple cell death pathways. During viral infection, host cells resist pathogens by activating PANoptosis. For example, after a virus invades a cell, the PRRs within the cell recognize the virus’s nucleic acids or proteins, activating inflammasomes (such as the AIM2 inflammasome) and cell death signaling pathways. These signaling pathways further activate key proteins such as members of the caspase enzyme family, RIPK1/RIPK3, and GSDMD, leading to apoptosis, necroptosis, and pyroptosis in the cell. This multi-pathway cell death method can effectively eliminate infected cells and prevent the further spread of the virus, thereby enhancing the host’s antiviral defense capabilities. However, excessive PANoptosis can also trigger excessive inflammatory responses and tissue damage, causing harm to the host. Therefore, the role of PANoptosis in viral infection is a double-edged sword, as it can aid in host defense but may also lead to pathological damage [25].

However, it is noteworthy that inhibiting PANoptosis exerts dual effects on host antiviral responses, with its specific impact depending on the pathogen type, infection stage, and host immune status. In some scenarios, PANoptosis inhibition may enhance host antiviral defense mechanisms, while in others, it could potentially exacerbate inflammatory responses and tissue damage. Therefore, when developing PANoptosis-targeted antiviral therapeutic strategies, these factors must be carefully considered to achieve optimal treatment outcomes.

In summary, through in-depth analysis of the pathological mechanisms of viral pneumonia, we have successfully developed an innovative lung-targeting inflammation-responsive nanodrug. This sophisticated nanoplatform integrates MA and Se with exquisite design: MA exerts its outstanding antiviral activity to precisely inhibit viral replication in pulmonary cells, while Se potently scavenges excess ROS through its robust antioxidant capacity, effectively suppressing oxidative stress. This dual-functional combination achieves synergistic antiviral and antioxidant effects. Comprehensive safety evaluations have confirmed the excellent biosafety profile of this nanodrug. Mechanistically, we pioneered a novel research paradigm by investigating PANoptosis inhibition. Using systematic cellular assays and animal infection models, we demonstrated that the nanodrug effectively modulates the ZBP1-RIPK3-MLKL-caspase signaling axis, significantly inhibiting apoptosis, necroptosis, and pyroptosis. Consequently, it blocks PANoptosis activation, reduces the excessive release of inflammatory cytokines and DAMPs, and thereby alleviates pulmonary inflammation and tissue damage. These findings provide groundbreaking theoretical evidence for elucidating the therapeutic mechanisms of MA + SeNPs against viral pneumonia.

## Ethical Approval

All animal studies were approved by the Ethics Committee of Zunyi Medical University (permit no. ZMU24-2408-09) and performed in accordance with the Laboratory Animal Welfare and Ethics Committee of China.

## Data Availability

All data of this study are included in the paper.
